# A New Biorecognition-Element-Free IDμE Sensor for the Identification and Quantification of *E. coli*

**DOI:** 10.3390/bios12080561

**Published:** 2022-07-25

**Authors:** Yung-Kai Lin, Hsing-Ju Wu, Nguyen Van Hieu, Pei-Yi Chu, Thi Vien Thao Do, Fiona Yan-Dong Yao, Thien Luan Phan, Congo Tak Shing Ching

**Affiliations:** 1 Institute of Food Safety and Risk Management, National Taiwan Ocean University, Keelung City 202, Taiwan; yklin@ntou.edu.tw; 2 Department of Food Science, National Taiwan Ocean University, Keelung City 202, Taiwan; 3 Graduate Institute of Biomedical Engineering, National Chung Hsing University, Taichung City 402, Taiwan; vienthao1310@gmail.com; 4 Research Assistant Center, Show Chwan Memorial Hospital, Changhua City 500, Taiwan; hildawu09@gmail.com; 5 Department of Medical Research, Chang Bing Show Chwan Memorial Hospital, Lukang Township 505, Taiwan; 6 Department of Physics and Electronic Engineering, University of Science (Vietnam National University of Ho Chi Minh City), Ho Chi Minh City 700000, Vietnam; nvhieu@hcmus.edu.vn; 7 Department of Pathology, Show Chwan Memorial Hospital, Changhua City 500, Taiwan; chu.peiyi@msa.hinet.net; 8 School of Medicine, College of Medicine, Fu Jen Catholic University, New Taipei City 242, Taiwan; 9 Division of Science, Engineering and Health Studies, The Hong Kong Polytechnic University-Hong Kong Community College, Hong Kong; fiona.yao@cpce-polyu.edu.hk; 10 Department of Electrical Engineering, National Chi Nan University, Puli Township 545, Taiwan

**Keywords:** *Escherichia coli*, interdigitated microelectrodes, biorecognition element free, impedance, capacitance

## Abstract

The label-free biosensor has emerged as an effective tool for the purpose of early detection of causative pathogens such as *Escherichia coli* as a preventive measure. In this study, a biorecognition-element-free interdigitated microelectrode (IDμE) sensor is designed and developed with this in mind, with good reliability and affordability. Results show that the designed sensor can identify *E. coli* with good selectivity using an impedance and capacitance of 7.69 MHz. At its optimum impedance of 1.3 kHz, the IDμE sensor can reliably quantify *E. coli* in a range of measurement (10^3.2^~10^6^ cfu/mL), linearity (R^2^ = 0.97), sensitivity (18.15 kΩ/log (cfu/mL)), and limit of detection (10^3.2^ cfu/mL). In summary, the IDμE sensor developed possesses high potential for industrial and clinical applications.

## 1. Introduction

The Centers for Disease Control and Prevention (CDC, US) reports that *E. coli O157:H7*, various strains of *Salmonella*, and *Listeria monocytogenes* were sources of food contamination [1], resulting in infections transmitted through contaminated fruit (apple), vegetables (lettuce, sprouts), drinking water, dairy product, meat, poultry, or improper food preservation [2,3,4,5,6]. These potentially entail economic costs in production and market loss and medical costs. The World Health Organization (WHO) estimated that the human cost of foodborne diseases in Vietnam alone exceeds US$1 billion a year, and this is about 2% of the gross domestic product (GDP) [7]. A study by Schrarff [7] introduced a model for foodborne diseases, which estimated that the average cost associated with each case of foodborne illness was US$1626 and US$77.7 billion annually in the United States. Although this model for the US is not applicable to low- and middle-income countries, it can be used as an indicator of the heavy economic burden of foodborne diseases.

Most microorganisms play an important role in nature while some are harmful to humans and animals in contaminated food and water. *E. coli* is a bacterium that is commonly found in the gut of humans and warm-blooded animals. Most *E. coli* are harmless, but some strains, such as Shiga toxin-producing *E. coli* (STEC), can cause severe foodborne diseases. *E. coli O157:H7*, the most important STEC serotype in public health, produces toxin that damages the intestine lining and causes anemia, stomach cramps and bloody diarrhea, and even serious hemolytic uremic syndrome (HUS) and thrombotic thrombocytopenic purpura (TTP) [8,9,10,11,12], with a greater effect on children, pregnant women, elderly, and the immune-impaired. Therefore, it is of great importance to develop novel, advanced, and efficient tools for the detection of foodborne pathogenic bacteria.

Microbiological culture techniques are considered the gold standard for sensitive and simple detection of bacteria but are time-consuming (2–3 days for initial results, up to 7–10 days for confirmation) and labor-intensive [13,14,15]. Polymerase chain reaction (PCR) is quicker (24 h including sample enrichment steps), highly specific and sensitive, accurate, and requires small samples [16]. Enzyme-Linked Immunosorbent Assay (ELISA) is another detection method that takes 48 h to assay with pre-enrichment to achieve the threshold limits for detection in food samples, with limits of 10^3^~10^5^ cfu/mL pathogens [17]. Both techniques have limitations that preclude their widespread implementation, including the need for special facilities, time-consuming enrichment steps, and a failure to discriminate between viable and non-viable cells [14,18]. As a result, conventional methods are inadequate for timely assessments of food safety.

Compared with conventional methods of biosensor technology, label-free methods are becoming popular due to their simplicity and ability to directly detect the target in real-time. The readout platforms commonly used are based on surface plasmon resonance (SPR), quartz crystal microbalance (QCM), or surface-enhanced Raman spectroscopy (SERS) [19,20,21,22,23,24]. Electrochemical impedance biosensors have been studied for qualitative and quantitative detection and monitoring of bacteria by means of the change impedance with captured bacteria in the medium or on electrodes [25]. Compared with other techniques in the family of electrochemical sensing such as voltametric and potentiometric, the information content of electrochemical impedance spectroscopy (EIS) is much higher than the DC technique or single-frequency measurements. Due to shifts in the applied frequency, EIS may be able to distinguish between two or more electrochemical reactions taking place and provide information on the capacitive behavior of the system.

The EIS technique involves exciting target samples with a weak sinusoidal electrical signal in a wide range of frequencies. The impedance responses are collected and correlated with changes in the samples [26]. However, despite the advantages of their label-free nature, these biosensors still rely on biorecognition elements, which presents challenge to mass production [27]. To overcome this limitation, an immobilization and biorecognition-element-free detection method would bring new hope for effective biosensor application in the food industry.

The EIS technique involves the use of electrodes. Previous studies showed that signal detection using electrodes with the same surface area but different shapes varied, with interdigitated electrodes having the highest sensitivity [28,29]. In addition, changing the shape of the electrode and increasing the edge length of the electrode were found to improve the sensor’s sensitivity [28,29]. Moreover, in these studies, gold (Au), silver (Ag), copper (Cu), etc. were common electrode materials, and gold was most favored due to its stability as an inert metal. Laibinis et al. used Au, Ag, and Cu as electrode materials and demonstrated that Au has the most advantages [30,31], including the ability to provide a friendly and efficient platform to immobilize enzymes and further improve electron transfer between active sites and the electrode. Moreover, gold is a highly biocompatible and high surface energy material, thus the enzyme keeps its biological activity and the loading of the enzyme increases. The diffusion rates affect the magnitude of the signal greatly; a gold surface with high biocompatibility allows the attached enzyme to have more freedom of orientation and weakens the insulating protein layer covering the active site.

Based on the research literature [28,29,30,31], gold interdigitated electrodes can help to improve the sensitivity and stability of sensors [32,33,34,35]. Therefore, the integration of different techniques was proposed, such as impedance measurement with gold interdigitated microelectrodes, to be important to the development of new impedance biosensors for the detection of pathogenic bacteria.

In this work, the development of a biorecognition-element-free impedance-capacitance sensor is reported, which is capable of detection and accurate identification of *E. coli O157:H7* down to 10^3^ cfu/mL (limit of detection: 10^3.2^ cfu/mL) in pure samples using an interdigitated microelectrode. The changes in the impedance and capacitance were detected using a precision impedance analyzer. It is sensitive, selective, economical, and does not require pre-enrichment steps. This sensor offers great potential for future studies and applications for the detection of bacteria.

## 2. Materials and Methods

### 2.1. Sample Preparation

All the solutions were prepared with double deionized (DDI) water (18.2 MΩ) produced by Elga CLXXXDIM2 Purelab Water Purification System, (Elga, USA). Samples of *E. coli O157:H7* and *Salmonella Typhimurium* were obtained from Department of Biotechnology, Asia University (Taichung, Taiwan), and stored at 4 °C before being revived at room temperature for testing.

*E. coli* and *Salmonella* bacteria were serially diluted in DDI water to obtain samples of different concentrations for subsequent use in impedance-capacitance spectrum measurements. Sample preparations were performed at room temperature (24 °C) at concentrations between 10^3^ and 10^6^ cfu/mL in 2-mL sterile centrifuge tubes.

### 2.2. Impedance Analyzer and IDμE Sensor

The impedance and capacitance spectrum analyses were conducted using a Wayne Kerr 6420C Impedance Analyzer (Wayne Kerr Electronics, UK). A 100-mV alternating potential was applied to measure the impedance (Z), capacitance (C), and phase angle (θ) in the frequency range of 20 Hz to 10 MHz. Cole–Cole plots and an equivalent circuit were used to interpret and present data.

**Figure 1:**
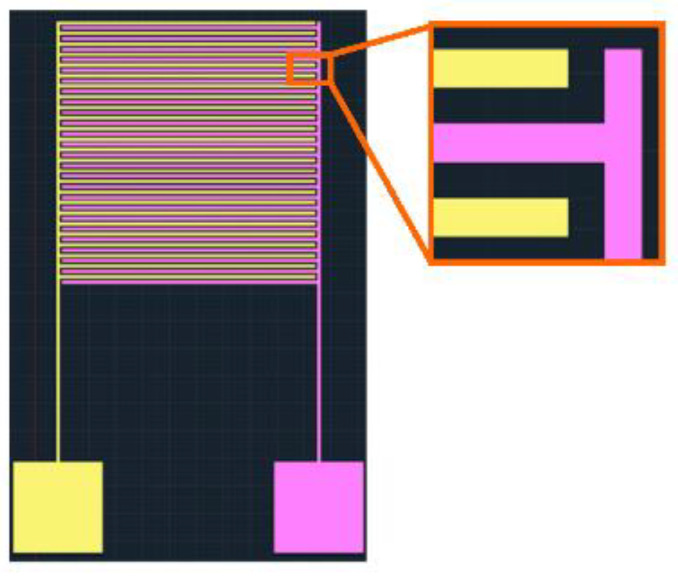
The configuration of the IDμE sensor.

**Figure 2:**
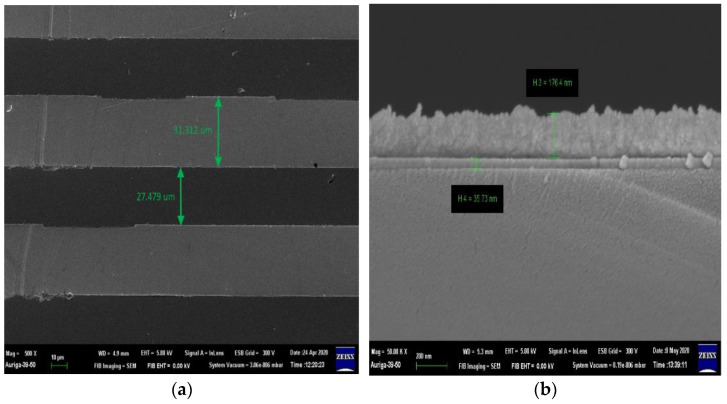
SEM images of the IDμE sensor. (**a**) Top plane, (**b**) side tomography.

The IDμE sensor mask design is important for this method. The configuration of the IDμE sensor and the electric connection is shown in Figure 1. The IDμE sensor includes 25 pairs of 30-μm-width gold finger, with a 30-μm inter-digit spacing and 3060-μm horizontal. The total working area of the IDμE sensor is about 6.92 mm^2^. Scanning electron microscope (SEM) images were taken to confirm the configuration of the IDμE sensor (Figure 2a).

A thin layer of titanium (Ti) was sputtered on to a Silic substrate to serve as an adhesive layer for the gold (Au) layer. The ratio between Ti:Au was set to a standard 1:3 ratio. An SEM image was also taken to confirm the thickness (Figure 2b).

### 2.3. Experimental Setup and Impedance-Capacitance Spectrum Measurements of the Pathogen Samples

**Figure 3:**
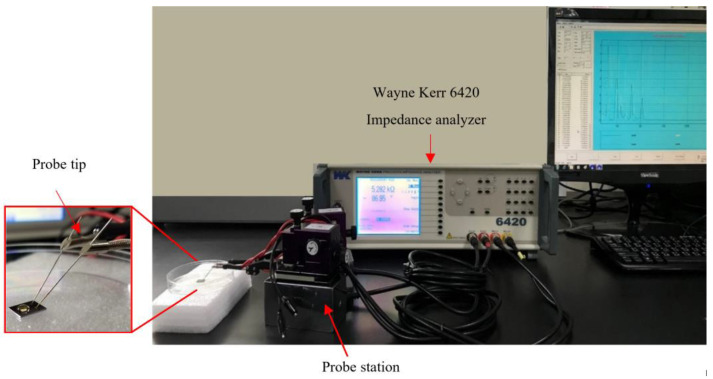
Experimental setup for impedance-capacitance spectrum measurements.

Bacteria detection at different sample concentrations was based on impedance-capacitance analysis in this study. This experimental setup consists of a probe station fixed on an adjustable metal block positioned between the tip and the IDμE sensor and an impedance analyzer for recording the signals (Figure 3). The measured data were stored on and printed out from a computer.

Before testing, the IDμE sensor was immersed in 75% ethanol, cleaned by the ultrasonic for 30 s, and then followed by DDI water washout, before being air dried in nitrogen. The IDμE sensor was then positioned under the impedance analyzer probe and the probe station adjusted so that the probe tip was firmly in contact with the electrodes of the IDμE sensor. A sample ($12^{-3}$ mL, concentration from 0 to 10^6^ cfu/mL) was pipetted onto the sensing window (interdigitated fingers) of the IDμE sensor to measure the Z, C, and θ spectra. Samples were tested in the order of 10^3^, 10^3.5^, 10^4^, 10^4.5^, 10^5^, 10^5.5^, and 10^6^ cfu/mL. Between measurements, a cleaning step was carried out by removing test solution and using DDI water and blotting paper. The measurement procedure was applied for both the *E. coli* and *Salmonella* samples.

### 2.4. Evaluation of the Biorecognition-Element-Free IDμE Sensor

Several tests were conducted to evaluate the performance of the IDμE sensor, including an optimum measuring frequency test, a linearity test, a sensitivity test, a quantification test, and an identification test, to determine the ability of the IDμE sensor to detect the pathogen.

## 3. Results and Discussion

### 3.1. Identification Test of the Biorecognition-Element-Free IDμE Sensor for the Detection of E. coli

**Figure 4:**
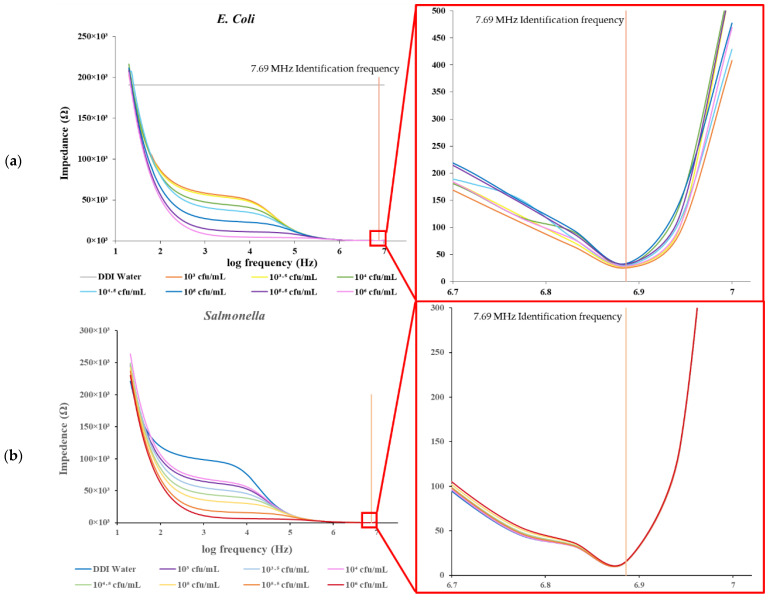
Impedance spectrum of (**a**) *E. coli* and (**b**) *Salmonella* samples.

**Figure 5:**
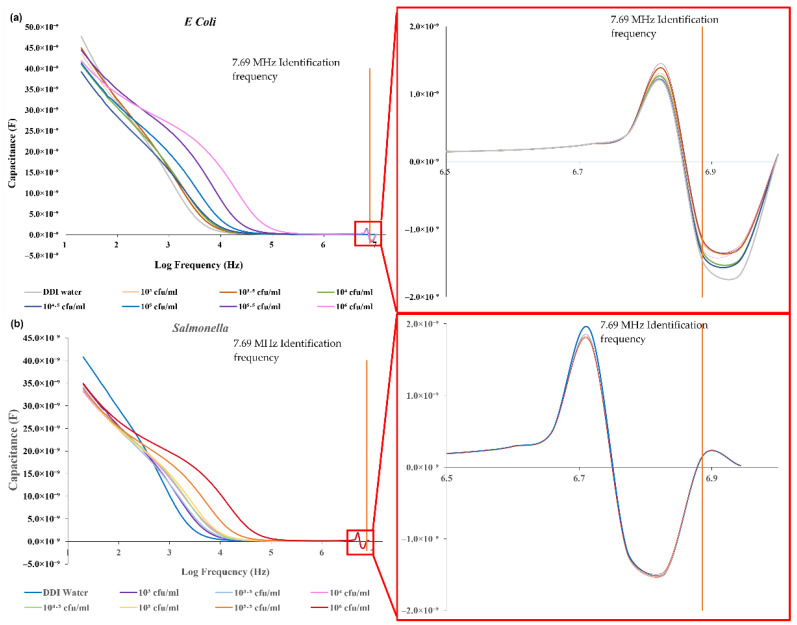
Capacitance spectrum of (**a**) *E. coli* and (**b**) *Salmonella* samples.

**Figure 6:**
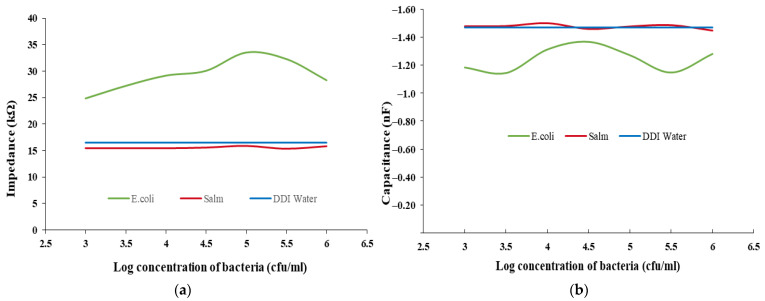
Differences in the (**a**) impedance and (**b**) capacitance between *E. coli* and *Salmonella* samples with NC at the characteristic frequency of 7.69 MHz.

**Figure 7:**
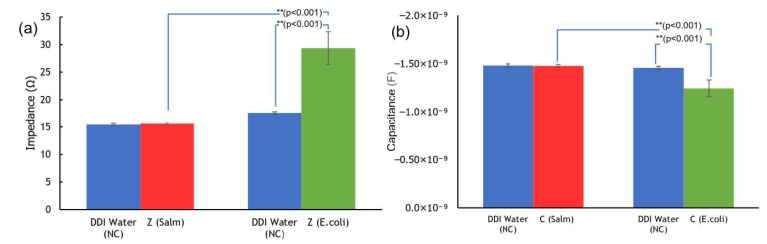
Mean value of the (**a**) impedance and (**b**) capacitance measurement data of *E. coli*, *Salmonella*, and their NC at the characteristic frequency of 7.69 MHz. Remarks: ** significant at *p* < 0.001.

Figure 4 and Figure 5 show the measurements of the impedance and capacitance from the *E. coli* and *Salmonella* bacteria samples at concentrations from 10^3^ to 10^6^ cfu/mL. In the data plot, special turning points are observed on both the impedance and capacitance curves at a frequency of 7.69 MHz, at which the data of *Salmonella* are similar to the values of the DDI water used as a negative control (ND), unlike the measurements from *E. coli*, which are significantly different (see Figure 6 and Figure 7).

As shown in Figure 6a, at 7.69 MHz, the impedance measurements of *Salmonella* and DDI water (as NC) are similar, at around 15 Ω, while the impedance of *E. coli* varies from 25 to 30 Ω. A similar behavior is observed in the capacitance values. The measurement of *Salmonella* is similar to NC at −1.48 nF while the *E. coli* responses vary from −1.4 to −1.1 nF (Figure 6b). The difference between the reading of DDI water, *E. coli*, and *Salmonella* could be caused by the error in the structure and configuration between the different electrodes; however, it can be neglected as the impedance change is approximately only 2 Ω and the capacitance change is less than 0.02 nF. A normal distribution test was performed on the *E. coli* and *Salmonella* samples with densities ranging from 10^3^ to 10^6^ cfu/mL, and the *p*-value was found to be 0.999 and 0.135, respectively (*p* > 0.05).

An independent T-test was carried out on the impedance data of *E. coli*, *Salmonella*, and NC samples, which identified significant differences (*E. coli* vs. *Salmonella* and *E. coli* vs. NC: both with *p* < 0.001) (Figure 7a). However, no significant difference was found between the impedance data of *Salmonella* and NC.

An independent T-test on the capacitance data of the E. coli, Salmonella, and NC samples found significant differences (*E. coli* vs. *Salmonella* and *E. coli* vs. NC: both with *p* < 0.001) (Figure 7b). Again, no significant differences existed between the capacitance data of the Salmonella and NC samples. The findings support the use of the IDμE sensor to measure the impedance and capacitance of samples and help to identify the presence of *E. coli* with good selectivity at a characteristic frequency of 7.69 MHz.

### 3.2. Optimum Measurement Frequency, Linearity, and Repeatability Test of the Biorecognition-element-Free IDμE Sensor for the Detection of E. coli O157:H7

The repeatability of the IDμE sensor was determined, and the results are a maximum and minimum relative standard deviation (RSD) of 19% and 3%, respectively (shows Table 1).

**Figure 8:**
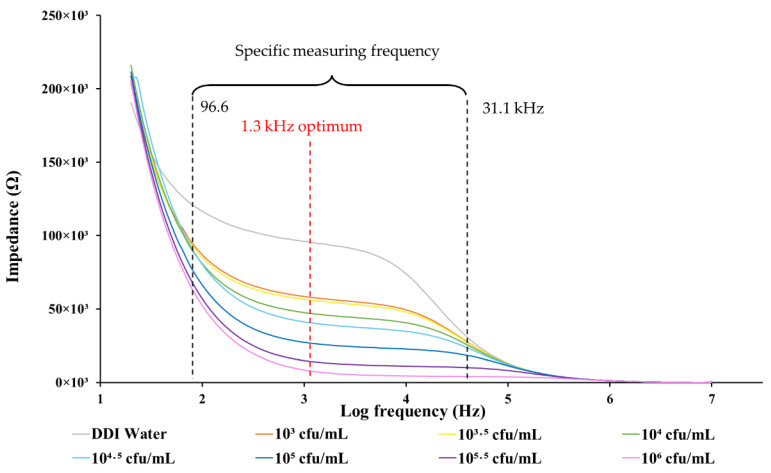
The spectrum of the impedance response of the IDμE sensor to various concentrations (10^3^ to 10^6^ cfu/mL) of *E. coli* samples. The optimal measurement frequency (1.3 kHz) and a specific measurement frequency range (96.6 Hz–31.1 kHz).

The impedance measured by the IDμE sensor in the frequency range between 20 Hz and 10 MHz on samples of *E. coli* at various concentrations is shown in Figure 8. In the specific measuring frequency range of 96.6 Hz–31.1 kHz, the data shows great linearity, with a coefficient of determination (R^2^) between the impedance response of the IDμE sensor and the logarithmic concentration of the *E. coli* samples greater than 0.9. The impedance of *E. coli* in the DDI water samples decreases as the *E. coli* concentration increases, indicating the impedance change correlates well with the concentration of *E. coli*, ranging from 10^3^ to 10^6^ cfu/mL.

**Figure 9:**
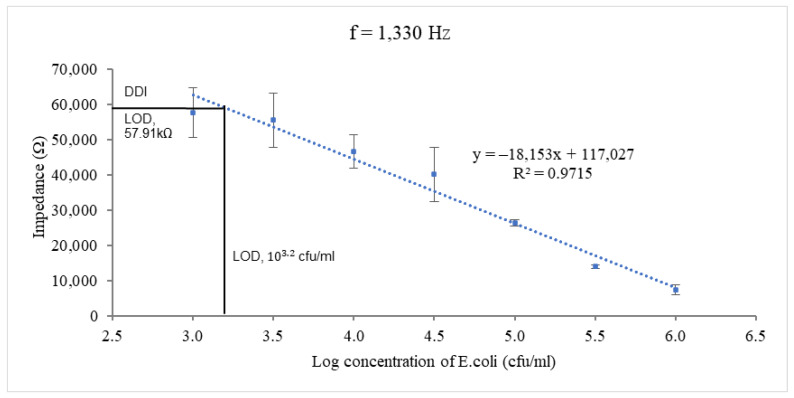
Calibration curves of the impedance magnitude of *E. coli* samples at different concentrations tested at the optimum frequency of 1.3 kHz. The limit of detection was determined by means of NC − 3 x standard deviation of NC. R^2^ is the coefficient of determination between the sensor’s impedance response and the logarithmic concentration of *E. coli*.

The linear relationship between the impedance magnitude and different logarithmic concentrations of *E. coli* at the optimum frequency of 1.3 kHz is shown in Figure 9. The optimal frequency refers to the frequency at which the IDμE sensor exhibited the greatest sensitivity with good linearity to the concentration of pathogens (R^2^ > 0.9). As shown in the figure, the impedance response fitted a simple linear regression line well against the sample concentration of *E. coli* ranging from 10^3^ to 10^6^ cfu/mL (R^2^ = 0.97), with a maximum sensitivity of 18.15 kΩ/log (cfu/mL), as determined by the slope of the fitting equation. This linear relationship between the impedance and concentration of bacteria is negative in nature. So, the limit of detection (LOD) was determined by the mean of the NC (94.8 kΩ) minus its standard deviation (12.2 kΩ) multiplied by 3 (S/N = 3). The result is 57.91 kΩ. Based on the calibration curves, the LOD is 10^3.2^ cfu/mL (or 1585 cfu/mL).

### 3.3. Equivalent Circuit Model for the Interpretation of the IDμE Sensor

**Figure 10:**
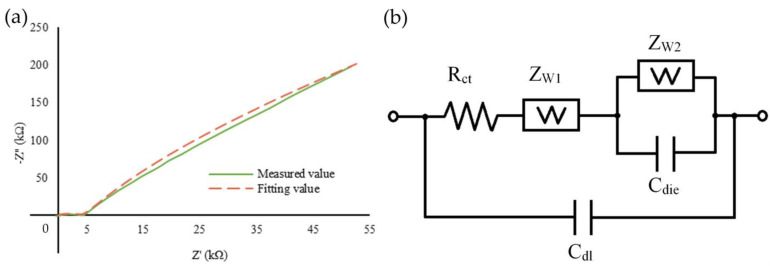
The “Cole–Cole plot” of the impedance and the fitted curve at 10⁶ cfu/mL of *E. coli* (**a**); equivalent Randle’s circuit representing the electrochemical system of the IDμE sensor (**b**).

The data and the fitted curve of the real and imaginary part of the impedance values from using the IDμE sensor to detect *E. coli* at 10^6^ cfu/mL are shown in Figure 10a. The fitting values overlap the measured values, indicating that the measured data can be expressed as an electrical circuit. Figure 10b shows the equivalent circuit generated by the CHI6378d Electrochemical Analyzer system, which represents the electrochemical system established in the sensor. It consists of R_ct_, Z_W1_, C_dl_, Z_W2_, and C_die_, where Z_W_ is the Warburg impedance representing the interfacial diffusion impedance; R_ct_ represents the charge transfer resistance, which is offered by the sensor construct since the opposition found by the electron mediators is due to the surface components; C_dl_ represents the double-layer capacitance generated by the ionic molecules in the sample overlaid near the surface of the electrode; and C_die_ is the dielectric capacitance, which is determined by the composition of the electrode. The value of the components in the mentioned equivalent circuit is shown in Table 2 for concentrations of 10^3^, 10^4^, and 10^6^. Typically, the dielectric capacitance (Cdie) constant depends on the insulation medium between the conducting plates. If this constant changes, meaning that the space medium has been changed, it is firmly believed that this change is due to the change in the concentration of the analyte.

### 3.4. Comparison of Different Sensors for E. coli Detection

A simple comparison of our biorecognition-element-free IDμE sensor with other sensors for the detection of *E. coli* shows that our sensor has an LOD between the LOD reported by Xu et al. (2016) and Lamanna et al. (2020). In fact, the commercially available BAX PCR system showed an LOD of 10^4^ cfu/mL, which required a detection time of 24 to 26 h [36]. All of these data confirm our designed IDμE sensor has an acceptable LOD and the advantages of reusability and ultrafast detection (Table 3).

## 4. Conclusions

In the present study, a biorecognition-element-free IDμE sensor was developed for the detection of *E. coli* in food samples. The sensitivity and selectivity of the sensor were determined using impedance and capacitance measurements of pure *E. coli* samples with different concentrations. The results indicate that the developed sensor is capable of specific detection of *E. coli* at a concentration of 10^3.2^~10^6^ cfu/mL with an LOD of 10^3.2^ cfu/mL in the pure samples. The changes in the impedance and capacitance at the characteristic frequency of 7.69 MHz were used to determine the existence and quantity of *E. coli* in the samples. The IDμE sensor possesses good sensitivity, high selectivity, affordability, and reusability (simple cleaning with ethanol 75% and deionized water). This shows great potential for antibody-free and close-to-real-time identification of *E. coli*.

## Figures and Tables

**Table 1:** The repeatability of the IDμE sensor on the *E. coli* sample with concentrations ranging from 10^3^ to 10^6^ cfu/mL at an optimal measurement frequency of 1.3 kHz.

*E. coli* Concentration (cfu/mL)	Mean (kΩ)	SD (kΩ)	RSD (%)
0 (DDI water)	94.76	12.28	13
10^3^	57.57	7.05	12
10^3.5^	55.55	7.69	14
10^4^	46.57	4.75	10
10^4.5^	40.10	7.74	19
10^5^	26.32	0.81	3
10^5.5^	13.92	0.46	3
10^6^	7.36	1.41	19

**Table 2:** Simulated data of the component in the equivalent circuit for the *E. coli* sample at different concentrations.

*E. coli* Concentration	R_ct_ (kΩ)	Z_W1_ (µΩ)	C_dl_ (nF)	Z_W2_ (µΩ)	C_die_ (nF)
$10^{3}$	52.04	1.74	0.29	0.12	50
$10^{4}$	41.41	1.79	0.25	0.13	42
$10^{6}$	3.83	5.86	0.32	0.13	32

**Table 3:** Comparison of other sensors developed to detect food pathogens with our IDμE sensor.

Biorecognition Elements	Target Bacteria	LOD (cfu/mL)	Detection Time (min)	Reusable	Reference
Needed	*E. coli*	$10^{2}$	105	No	[37]
Needed	*E. coli*	$10^{5}-10^{6}$	90	No	[38]
Needed	*E. coli*	$2.05\;\times \;10^{3}$	NA	Yes	[33]
Needed	*E. coli*	$10^{6}$	NA	No	[39]
Needed	*E. coli*	$10^{3}$	60	No	[40]
No need	*E. coli*	$10^{3.2}$	2~3	Yes	Our sensor

NA: not available.

## Data Availability

Not applicable.
